# Heparin-Induced Anaphylaxis With Cardiac Arrest in a Patient With Alpha-Gal Syndrome: A Case Report

**DOI:** 10.7759/cureus.110413

**Published:** 2026-06-07

**Authors:** Ramtin Khanipour, Reetom K Bera, Idelisse Suarez, Arman Soltani Moghadam, Jishanth Mattumpuram

**Affiliations:** 1 Internal Medicine, HCA Florida Bayonet Point Hospital, Hudson, USA; 2 Tehran Heart Center, Cardiovascular Diseases Research Institute, Tehran University of Medical Sciences, Tehran, IRN; 3 Cardiology, University of Louisville Hospital, Louisville, USA

**Keywords:** alpha-gal syndrome, cardiac arrest, case report, heparin, ventricular fibrillation

## Abstract

Alpha-gal syndrome (AGS) is a delayed allergic reaction to red meat linked to bites of various species of ticks around the world, with the common culprits in North America identified as the Ixodes and Lone Star ticks. Most cases are asymptomatic, but some may result in mild allergic reactions or could progress to severe anaphylaxis.

This report presents the case of a 64-year-old male with a history of coronary artery disease who presented with chest pain. During his hospitalization, he experienced two episodes of ventricular fibrillation (VF) with successful resuscitation. Following discharge, he continued to experience episodic palpitations, abdominal pain, nausea, vomiting, and diarrhea. An immunologist later diagnosed him with AGS and introduced the possible association of the VF events with a severe hyperacute reaction to intravenous heparin exposure, either in conjunction with or independent of the STEMI the patient was experiencing. His chronic longitudinal symptoms resolved entirely with dietary restrictions, specifically avoiding beef and porcine meat derivative products.

This report highlights the importance of considering AGS in patients with unexplained allergic or periprocedural reactions following exposure to mammalian-derived products such as heparin.

## Introduction

Alpha-gal syndrome (AGS) is an immunoglobulin E (IgE)-mediated hypersensitivity to galactose-α-1,3-galactose (alpha-gal), a carbohydrate found in non-primate mammals, following sensitization via tick bites (e.g., Ixodes or Lone Star ticks). Through natural selection, the alpha-gal coding region has been terminally inactivated in the human genome as well as other primates and Old World monkeys but remains abundant in other mammals. Modern humans produce antibodies against the α-Gal oligosaccharide [1]. Therefore, the human immune system generates an immunological reaction when exposed to it [2].

AGS is commonly reported in the southeastern United States, Europe, and Australia, where it mostly affects the adult population. Alpha-gal exposure occurs through the consumption of mammalian products or medications containing galactose-α-1,3-galactose antigen in their chemical composition [3]. Since most medications containing alpha-gal are administered intravascularly, they can rapidly evoke an allergic reaction when compared to alpha-gal-containing food products [4]. Heparin is an alpha-gal-containing medication, as are anti-snake venom therapies, certain bovine or porcine-derived thyroid hormone supplements, any medications with gelatin components (like many vaccines), and cetuximab [4].

Exposure may be asymptomatic or produce symptoms such as pruritus, urticaria, angioedema, abdominal pain, nausea, vomiting, weight loss, joint pain, and, in severe cases, anaphylaxis. While most cases may feature delayed mild allergic symptoms upon oral exposure to products, a minority of patients can rapidly develop serious reactions when antigen-containing medications are administered intravenously [5].

Therefore, a patient with a history of tick bites may develop a hypersensitivity to the alpha-gal antigen, producing hypersensitivity reactions with exposure to products containing alpha gal. The condition can remain insidious until an event of extreme exposure, such as the intravenous administration of medication containing alpha gal. This case presents the manifestation of AGS as a periprocedural ventricular fibrillation (VF) following intravenous heparin administration during STEMI management, highlighting a severe and underrecognized complication.

This article was previously presented as a meeting abstract at the 2025 European Society of Cardiology Congress on August 31, 2025.

## Case presentation

A 64-year-old male with a past medical history of coronary artery disease with four prior drug-eluting stents in the left anterior descending artery and right coronary artery, paroxysmal atrial fibrillation, type 2 diabetes mellitus, peripheral arterial disease, and hypertension presented to the emergency department (ED) complaining of new-onset chest pain and shortness of breath. He also stated that he had been experiencing episodic hives, lips and tongue swelling, chest pressure, abdominal pain, nausea, vomiting, and diarrhea chronically over the past few years. While in the ED, the patient was diagnosed with an ST-elevated myocardial infarction (STEMI) based on elevated troponin-T and creatine kinase-MB levels (0.23 ng/mL and 25.8 ng/mL, respectively) (Table 1), as well as electrocardiogram (EKG) findings of ST elevation in the anterior leads and reciprocal depressions in the inferior leads (Figure 1). A bolus of intravascular unfractionated heparin was administered, and shortly after, the patient became hypotensive and drowsy and developed VF cardiac arrest. Return of spontaneous circulation (ROSC) was achieved after one round of advanced cardiac life support (ACLS). Electrolyte levels were within the normal range. A STAT transthoracic echocardiogram revealed a preserved ejection fraction of 50-55% with no wall motion abnormalities. Emergent left heart catheterization identified a 90% stenotic lesion in the diagonal branch, which was revascularized with a single drug-eluting stent. Following the intervention, the patient was loaded with aspirin and clopidogrel, but since the activated clotting time was measured to be 230 seconds, he received an additional bolus of intravascular heparin, and once again, within 30 minutes, the patient became hypotensive and had his second VF cardiac arrest. After ROSC was achieved, heparin was discontinued, and he was transitioned to dual antiplatelet therapy. It is important to note that prior to the current events, the patient's last heparin exposure was 10 years earlier, with no adverse reactions or development of any arrhythmia. At discharge, the VF episodes were attributed to his acute myocardial infarction.

**Table 1:** Cardiac marker laboratory values at initial presentation to the emergency department.

Laboratory Test	Value	Reference Range
Troponin-T (ng/mL)	0.23	0-0.02
Creatine kinase - MB (CK-MB) (ng/mL)	25.8	0-7.7

**Figure 1:**
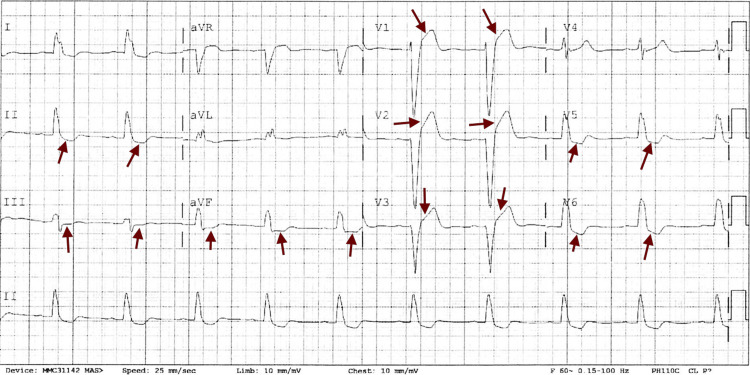
Presenting electrocardiogram with ST elevations in leads V1 through V3, reciprocal depressions in II, III, and aVF, and depressions in V5 and V6.

After discharge, the patient continued to experience palpitations, shortness of breath, tongue swelling, urticaria, abdominal pain, nausea, vomiting, and diarrhea, particularly after consuming red meat. He visited the ED multiple times over the next 18 months, but no precise diagnosis was confirmed. A gastroenterologist conducted an in-depth evaluation but found no clear etiology. He was subsequently referred to an allergist and immunologist who ordered allergen-specific IgE tests. Results were significant for an elevated serum IgE level against beef at 0.16 kU/L (Table 2). Given the patient's residential history in Kansas, where he had experienced numerous tick bites, an AGS assay was performed. Results confirmed the diagnosis of AGS with galactose alpha 1,3 galactose IgE level at 0.32 kU/L (Table 3).

**Table 2:** Allergen-specific IgE tests showing an elevated serum IgE level against beef (0.16 kU/L; normal <0.10 kU/L). IgE, immunoglobulin E

Laboratory Test	Value	Flag	Reference Value	Reference Class
IgE allergen panel values				
IgE oak, white (kU/L)	<0.10	-	<0.10	Class 0
IgE elm, American (kU/L)	<0.10	-	<0.10	Class 0
IgE pecan, hickory (kU/L)	<0.10	-	<0.10	Class 0
IgE ragweed, short (kU/L)	<0.10	-	<0.10	Class 0
IgE pigweed, common (kU/L)	0.14	Abnormal	<0.10	Class 0/I
IgE sheep sorrel (kU/L)	<0.10	-	<0.10	Class 0
IgE nettle (kU/L)	<0.10	-	<0.10	Class 0
IgE mouse urine (kU/L)	<0.10	-	<0.10	Class 0
IgE food profile with component reflex				
IgE egg white (kU/L)	<0.10	-	<0.10	Class 0
IgE milk (kU/L)	<0.10	-	<0.10	Class 0
IgE codfish (kU/L)	<0.10	-	<0.10	Class 0
IgE wheat (kU/L)	<0.10	-	<0.10	Class 0
IgE corn (kU/L)	<0.10	-	<0.10	Class 0
IgE sesame seed (kU/L)	<0.10	-	<0.10	Class 0
IgE peanut (kU/L)	<0.10	-	<0.10	Class 0
IgE soybean (kU/L)	<0.10	-	<0.10	Class 0
IgE shrimp (kU/L)	<0.10	-	<0.10	Class 0
IgE clam (kU/L)	<0.10	-	<0.10	Class 0
IgE walnut (kU/L)	<0.10	-	<0.10	Class 0
IgE scallop (kU/L)	<0.10	-	<0.10	Class 0
Allergen profile, food-meat				
IgE pork (kU/L)	<0.10	-	<0.10	Class 0
IgE beef (kU/L)	0.16	Abnormal	<0.10	Class 0/I
IgE chicken (kU/L)	<0.10	-	<0.10	Class 0
IgE garlic (kU/L)	<0.10	-	<0.10	Class 0
IgE onion (kU/L)	<0.10	-	<0.10	Class 0
IgE D pteronyssinus (kU/L)	<0.10	-	<0.10	Class 0
IgE farinae (kU/L)	<0.10	-	<0.10	Class 0
IgE cat dander (kU/L)	<0.10	-	<0.10	Class 0
IgE dog dander (kU/L)	<0.10	-	<0.10	Class 0
IgE Bermuda grass (kU/L)	<0.10	-	<0.10	Class 0
IgE Bahia grass (kU/L)	<0.10	-	<0.10	Class 0
IgE Timothy grass (kU/L)	<0.10	-	<0.10	Class 0
IgE cockroach, German (kU/L)	<0.10	-	<0.10	Class 0
IgE *Penicillium chrysogen* (kU/L)	<0.10	-	<0.10	Class 0
IgE *Cladosporium herbarum* (kU/L)	<0.10	-	<0.10	Class 0
IgE *Aspergillus fumigatus* (kU/L)	<0.10	-	<0.10	Class 0
IgE *Alternaria alternata *(kU/L)	<0.10	-	<0.10	Class 0
IgE maple/box elder (kU/L)	<0.10	-	<0.10	Class 0
IgE common silver birch (kU/L)	<0.10	-	<0.10	Class 0
IgE cedar, mountain (kU/L)	<0.10	-	<0.10	Class 0

**Table 3:** Alpha-gal panel showing an elevated serum IgE level against galactose alpha-1,3-galactose (0.32 kU/L; normal <0.10 kU/L), confirming the diagnosis of alpha-gal syndrome. IgE, immunoglobulin E

Laboratory Test	Value	Reference Range
Galactose alpha 1,3 galactose IgE	0.32 kU/L	<0.10 kU/L

To prevent disease progression and recurrence of symptoms, the patient was recommended lifestyle changes to avoid exposure to known triggers. In response, the patient worked to eliminate beef and pork products from his diet entirely. Upon the elimination of his known triggers, the patient reported resolution of his acute episodic symptoms. Furthermore, the patient experienced improvement and recovery of his functional status over the course of 12 more months (Figure 2).

**Figure 2:**
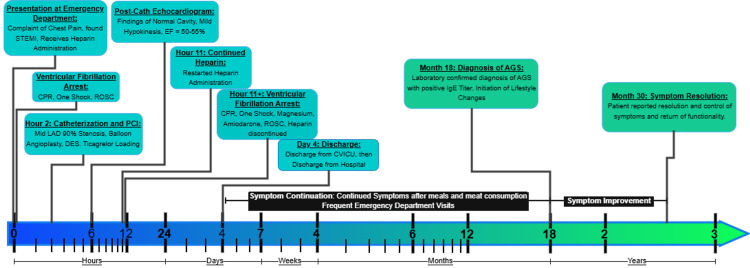
Visual timeline of the pathophysiology course from initial hospitalization through the workup and diagnosis of AGS to management and symptom resolution. The figure was created using draw.io (diagrams.net) and finalized using paint.net. AGS, alpha-gal syndrome; Cath, cardiac catheterization; CPR, cardiopulmonary resuscitation; CVICU, cardiovascular intensive care unit; DES, drug-eluting stent; EF, ejection fraction (left ventricular); IgE, immunoglobulin E; LAD, left anterior descending artery; PCI, percutaneous coronary intervention; ROSC, return of spontaneous circulation; STEMI, ST-elevated myocardial infarction

## Discussion

AGS is an acquired hypersensitivity originating from a bite from various species of ticks around the world, where the mammalian membrane oligosaccharide galactose-α-1,3-galactose (alpha-gal) can be introduced to the bloodstream during feeding (Figure 3). This eventually produces a sensitization to the alpha-gal antigen through IgE antibody interaction with mast cells and basophils in the recipients of the bites. Subsequent exposure to alpha-gal antigens often delivered through consumption of mammalian products results in the IgE antibody response to alpha-gal antigen, resulting in a hypersensitivity reaction.

**Figure 3:**
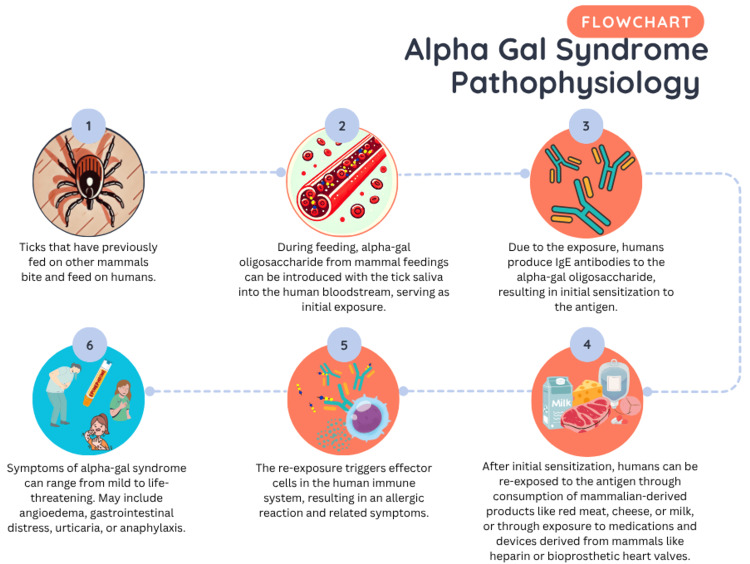
Pathogenesis of alpha-gal syndrome along with clinical manifestations. The figure was created using Canva IgE, immunoglobulin E

Our case report demonstrated that intravenous heparin exposure as part of the acute coronary syndrome protocol in a patient with confirmed AGS may precipitate reactions as extreme as fatal arrhythmia, such as VF cardiac arrest. Similarly, Muller et al. described a case where a patient with no significant past medical history presented with near cardiac arrest and persistent severe hypotension following the consumption of pork. The patient was found to have an elevated serum IgE against alpha-gal (0.85 kU/L) [6], further supporting the possibility of anaphylaxis-induced cardiac arrest in patients with AGS upon exposure to porcine-derived products. Furthermore, Hawkins et al. found that 50% of patients with AGS developed a severe allergic reaction to heparin during cardiopulmonary bypass [7].

In the patient presented in this report, it may stand to reason that intravenous exposure to porcine-derived heparin could have produced a hypersensitivity reaction severe enough to precipitate VF cardiac arrest. However, a notable limitation must be acknowledged that the patient was, on initial heparin exposure, concurrently experiencing a STEMI. It is well known in medical literature that cardiac arrest from ventricular tachyarrhythmias is a downstream complication of STEMI.

However, it has been demonstrated in numerous landmark studies that the reactive state from hypersensitivity can worsen myocardial ischemia. This is known as Kounis syndrome based on foundational work by Kounis, who demonstrated mast cell degranulation, and a resultant cascade including histamine, platelet-activating factor, and cytokines promoting coronary vasospasm, among other features of atherothrombotic events [8]. Quetsch et al. presented a 2025 systematic review of allergic vasospastic angina precipitating ACS events following allergen exposure [9]. It is likely that the VF arrest this patient experienced on initial exposure represents an acute worsening of an ongoing STEMI due to the allergic reaction to intravenous heparin.

The reaction following the subsequent exposure to intravenous heparin provides a basis for suspecting the hypersensitivity reaction to be a key contributor to the ventricular arrhythmia. He et al. demonstrated the role of histamine as a sympathetic neurotransmitter with arrhythmogenic effect inducing acute ischemia in mouse hearts. Histamine H2 receptor activation was associated with the development of ischemia-induced ventricular arrhythmias and was found to decrease in the presence of the selective H2 receptor antagonist famotidine, demonstrating that histamine facilitated the arrhythmogenic effect of simultaneously released norepinephrine [10]. Therefore, it stands to reason that, in the setting of a vulnerable myocardium from a recent acute coronary syndrome, the VF event in our patient was contributed to by the histamine release from hypersensitivity to heparin.

Now the question is what can clinicians do in the case of patients with a confirmed diagnosis of AGS who require anticoagulation? To answer this question, we reference a study conducted by Nwamara et al., in which allergic reactions to porcine derivative heparin in patients with AGS were assessed. Results indicated that 2.6% of patients with AGS experienced an allergic reaction when compared to unfractionated heparin, versus none on enoxaparin [11]. Additionally, Iglesia et al. demonstrated that sensitized patients have greater rates of reactions following intravenous heparin use compared to both subcutaneous heparin and subcutaneous enoxaparin. In a systematic review of multiple single-center data points, a reaction rate was observed in 4 (24%) of 17 patients receiving intravenous heparin, 1 (2.6%) of 39 receiving unfractionated subcutaneous heparin, and 0 (0%) of 22 receiving low-molecular-weight heparin [12]. In addition to its derivation from porcine products, an explanation as to why heparin is more likely to generate an immunological reaction is due to its larger molecular size relative to enoxaparin. This finding highlights the potential use of enoxaparin as an alternative to heparin in patients with AGS.

To tackle AGS in clinical practice, providers should raise awareness in tick-endemic areas and be vigilant in testing for galactose alpha-1,3-galactose IgE in patients experiencing unexplained allergic reactions. Furthermore, AGS itself can be investigated to generate risk stratification and improve early detection of the syndrome. While early detection may have utility in preventative care, utility of pre-procedural testing is likely to rest on the rapidity of the testing process itself. Pursuant to preventative care, improved early detection of AGS can lead to improvement in patient education and lifestyle modification, such as dietary avoidance. In cases of confirmed AGS, if heparin use is inevitable, a pre-medication regimen with corticosteroids, antihistamines, or omalizumab has been shown to reduce the frequency and severity of allergic reactions to heparin [13].

This case highlights the importance of considering AGS as a differential in patients with unexplained anaphylactic reactions, especially in those who may have been exposed to tick bites, limited primarily by a lack of documentation of severe arrhythmias and arrest and by uncertainties about AGS itself. Most documentation refers to severe allergic reactions from intravenous heparin largely in the setting of cardiac surgery. Our literature search has not uncovered any reports explicitly connecting heparin-triggered anaphylaxis to cardiac arrest. Instead, literature has separately identified the pathophysiology of AGS and hypersensitivity to mammalian-derived products, as well as links between hypersensitivity reactions and myocardial ischemia or acute coronary events. Complications of acute coronary syndrome, including ventricular arrhythmias and cardiac arrest, are also well documented. This case report may be a novel link between the hypersensitivity triggered by exposure to mammalian-derived products in patients sensitized through AGS and the triggering of or worsening of myocardial ischemia and acute infarction. This report also highlights the importance of individualized risk assessment in individuals at risk of AGS in a setting where they may require intravenous heparin, thereby calling for caution and consideration of alternative anticoagulants such as direct oral anticoagulants or synthetic heparin products in suspected AGS cases to prevent severe allergic reactions [14].

## Conclusions

AGS carries a risk of severe allergic reactions, including anaphylaxis, to mammalian products containing galactose alpha-1,3-galactose due to the IgE response primed by prior exposure to the carbohydrate from an Ixodes or Lone Star tick vector. As subsequent exposure can take the form of mammalian-derived medical products, awareness becomes essential for healthcare providers in the management of patients with a history of tick bites and unexplained hypersensitivity reactions. Education of both healthcare providers and patients is important to mediate reactions and prevent exacerbation of the disease through unregulated exposure (both environmental and nosocomial). Additionally, non-alpha-gal containing alternatives to medications ought to be considered if clinically efficacious. For example, enoxaparin or other synthetic anticoagulants are viable alternatives to porcine-derived heparin in AGS cases like the one described in this case report.

Currently, the overall collection of literature on AGS leaves room for additional reports and literature, and it appears that the overall consensus on the existence of and clinical significance of AGS is yet under much discussion. As such, this case report provides an additional account and further support toward the establishment and clinical significance of this condition and its implications in medical management. In conclusion, AGS is a lesser known but clinically important condition that should be taken into consideration by clinicians and patients alike for the purposes of early detection and preventative care and for its clinical implications in the use of known antigenic medical products.
